# Effect of domperidone (leisguard®) on antibody titers, inflammatory markers and creatinine in dogs with leishmaniosis and chronic kidney disease

**DOI:** 10.1186/s13071-021-05030-8

**Published:** 2021-10-10

**Authors:** Maria Alfonsa Cavalera, Floriana Gernone, Annamaria Uva, Paola D’Ippolito, Xavier Roura, Saverio Paltrinieri, Andrea Zatelli

**Affiliations:** 1 Department of Veterinary Medicine, University of Bari, Valenzano, Italy; 2 Veterinary Diagnostic Lab ACV Triggiano, Triggiano, Italy; 3 Hospital Clínic Veterinari, Universitat Autònoma de Barcelona, Bellaterra, Spain; 4 Department of Veterinary Medicine, University of Milan, Milan, Italy

**Keywords:** Antibody titer, Creatinine, CRP, Dog, Domperidone, Gamma globulins, Globulins, Leishmaniosis

## Abstract

**Background:**

Immunotherapeutic drugs, such as domperidone, have been shown to be promising treatments against canine leishmaniosis (CanL), but limited data are available. The aim of this pilot study (therapeutic, prospective and non-controlled) was to evaluate the effect of domperidone on serum antibody titers of* Leishmania infantum*, globulins, gamma globulins, acute-phase proteins (e.g. C-reactive protein [CRP]), big endothelin-1 (big ET-1), serum creatinine (SC) and proteinuria in dogs with leishmaniosis affected by chronic kidney disease (CKD).

**Methods:**

Dogs were recruited if “exposed” to or “infected” with *L. infantum* and affected by CKD (IRIS stage 1 [proteinuric] or IRIS stage 2–3a [SC < 3.5 mg/dl; proteinuric or non-proteinuric]). After inclusion, an oral suspension of domperidone was administered, and the dogs were followed up for 180 days, with checks at 30, 60, 90 and 180 days after initial treatment.

**Results:**

Of the 14 recruited dogs, nine showed a statistically significant reduction in SC (*χ*^2^ = 9.1, *df* = 3, *P* = 0.028), but not in the urine protein/creatinine ratio (*χ*^2^ = 6.43, *df* = 3, *P* = 0.092). All dogs showed a significant reduction in antibody titers for *L. infantum* (*χ*^2^ = 9.56, *df* = 2, *P* = 0.008), globulins (*χ*^2^ = 11.08, *df* = 3, *P* = 0.011) and gamma globulins (*χ*^2^ = 12.38, *df* = 3, *P* = 0.006) during the study period. There was also a statistically significant reduction in CRP (*χ*^2^ = 16.7, *df* = 3, *P* = 0.001), but not in big ET-1 (*χ*^2^ = 2.04, *df* = 3, *P* = 0.563).

**Conclusions:**

This study provides preliminary results on the ability of domperidone to improve SC and reduce anti-*L. infantum* antibody titers, globulins, gamma globulins and CRP in dogs with leishmaniosis and CKD.

**Graphical abstract:**

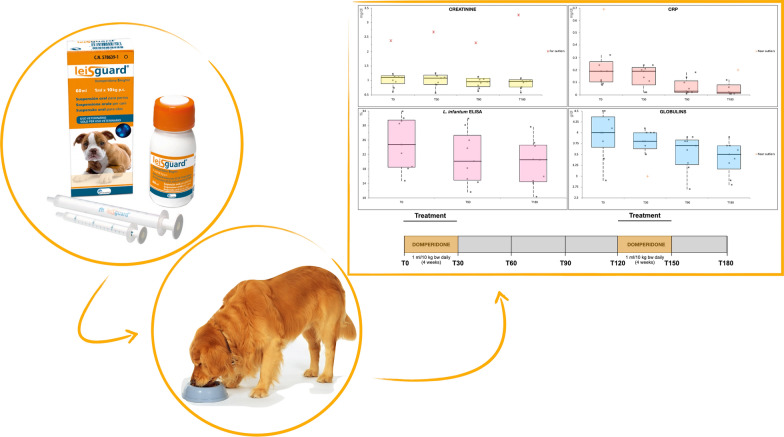

Canine leishmaniosis (CanL) is a major global sand fly-borne disease caused by *Leishmania infantum*, affecting at least 2.5 million dogs in southwestern Europe [1–3]. Although the high levels of seroprevalence by *L. infantum* in hyperendemic foci (e.g. 28.2% in southern Italy [4]), clinical disease affects only a limited proportion of infected dogs [5]. The different outcomes of leishmaniosis in canine populations, ranging from infection without clinicopathological changes to potentially fatal disease, depend mainly on the individual immune response of the affected dog. Indeed, in dogs susceptible to leishmaniosis, disease progression is due to both a marked humoral immune response and an impaired cellular immune response against the protozoa [2, 6].

The production of anti-*Leishmania* antibodies does not provide protection against CanL as it leads to hyperglobulinemia and the appearance of soluble circulating immune complexes (CIC) if an adequate antigen/antibody ratio, i.e. a moderate prevalence of antigens with respect to antibodies, is present [7–10]. Defective clearance of these CIC by scavenging macrophages induces their deposition in specific organs, such as in the kidney, resulting in proteinuric glomerulonephritis and, consequently, tubulo-interstitial lesions [11]. CIC-mediated renal pathology plays a pivotal role in the prognosis of CanL as chronic kidney disease (CKD) leading to severe renal damage represents the major cause of death in dogs with leishmaniosis [12]. Therefore, survival time and progression of CanL are strongly influenced by an early diagnosis and an appropriate therapeutic approach of infectious and renal diseases [12].

The first-line treatment protocol for dogs with leishmaniosis and kidney disease includes a leishmanicidal drug (i.e. meglumine antimoniate at 100 mg/kg twice daily or once daily subcutaneously for 1 month) in combination with a leishmaniostatic drug (i.e. allopurinol at 10 mg/kg orally twice daily or once daily together with xanthinuria, for at least 12 months) [13–15]. If this therapeutic regimen is not possible, the second-line treatment is a combination of miltefosine (2 mg/kg orally once daily for 28 days) and allopurinol [13, 14]. However, a parasitological cure (i.e. elimination of parasites from tissues) with the currently available compounds is rarely achieved, and clinical relapses can still appear weeks to years after the beginning of treatment [16–18].

In this scenario, despite the limited data that are currently available, immunotherapeutic treatments have shown to be promising against CanL, with the main objective of re-establishing dog immunity and, therefore, promoting parasite reduction and improving clinical signs [19–22]. Indeed, the use of non-specific immune modulatory treatments has been reported as potentiating the immune system of sick dogs to control the infection and to prevent the development of clinical disease in uninfected dogs [23–25].

Domperidone, an immunotherapy drug, has been shown to be useful for the management of the early stages of CanL or for the prevention of clinical disease as part of an integrated control program [15, 19, 26]. For example, in one study, domperidone was able to induce clinical improvement in 86% of the dogs affected by leishmaniosis with multiple clinical signs, with serum antibody titers decreased by 38% [19]. Indeed, domperidone enhances the cell-mediated immune response, potentiating the phagocytic and oxidative functions of canine neutrophils [20]. Domperidone is also a peripherally acting specific dopamine 2 (DA_2_) receptor antagonist [27], and evidence suggests that the intrarenal DA_2_ receptor in dogs plays a role in the control of renal function [28, 29]. Indeed, intrarenal administration of specific DA_2_ receptor antagonist increases glomerular filtration rate (GFR), renal plasma flow (RPF) and filtration fraction in uni-nephrectomized dogs [28], while intrarenal DA_2_ receptor stimulation decreases renal function by hemodynamic mechanisms [29].

Therefore, based on the considerations outlined above, the primary aim of this study was to evaluate the efficacy of domperidone (leisguard®; Ecuphar Italia srl, Milan, Italy) in: (i) maintaining and/or improving renal function (stable or decreased serum creatinine [SC]) and (ii) maintaining and/or reducing proteinuria (stable or decreased urinary protein/creatinine ratio [UPC]), in dogs with leishmaniosis affected by CKD. Moreover, in order to confirm previous published data [19, 20, 30], we also investigated the effect of leisguard® on serum antibody titers for *L. infantum* and on the concentrations of globulins, gamma globulins, C-reactive protein (CRP) and big endothelin-1 (big ET-1) in dogs with leishmaniosis.

This study was a therapeutic, prospective and non-controlled field trial conducted in two areas where CanL is endemic in southern Italy (i.e. Apulia and Basilicata regions) [4] from May to November 2018. Privately owned dogs of any sex, age, weight and breed were recruited for participation in this pilot study if classified as “exposed” to (i.e. stage A) or “infected” with (i.e. stage B) *L. infantum* according to the Canine Leishmaniasis Working Group (CLWG) staging system [2] and if affected by proteinuric CKD (UPC > 0.5) classified as IRIS stage 1 up to stage 3a or by non-proteinuric CKD (UPC ≤ 0.5) classified as IRIS stage 2 up to stage 3a (i.e. with SC < 3.5 mg/dl) [31].

Dogs were excluded if suspected or known to be affected by: (i) co-infections with other vector-borne pathogens, such as *Ehrlichia canis* and *Anaplasma phagocytophilum*; (ii) diseases able to determine progression of CKD and/or to increase the levels of of inflammatory markers (e.g. neoplastic, auto-immune and heart diseases, diabetes mellitus and insipidus, hypo- and hyperadrenocorticism or hyper- and hypothyroidism); (iii) active forms of CanL (i.e. stages C–D according to the CLWG staging system) [2]. All dogs that had been administered either leishmanicidal or leishmaniostatic treatments in the previous 6 months, as well as those that had also been in therapy for kidney disease for < 6 months or whose CKD was not stable, were also excluded [32].

At inclusion time (T0), each dog was physically examined, following which blood samples were collected from either the cephalic or jugular veins into K3 EDTA tubes (2 ml/tube) to undergo routine hematology and into plain tubes (5 ml/tube) to obtain serum after centrifugation (15 min at 1500 *g*). Urine samples were collected by free catch or cystocentesis [33] and put into sterile, evacuated, 10-ml collection tubes; these were stored at room temperature (approx. 20 °C [67.6 °F]) and analyzed within 4 h from collection. For each enrolled dog, a complete blood count (CBC) with reticulocyte count, a complete biochemical panel, including APPs (i.e. CRP), big Et-1, electrophoresis and fibrinogen concentration measurements, and a complete urine exam with osmolality and UPC were performed. Furthermore, at T0, T90 (follow-up day 90) and T180 (follow-up day 180), the dogs were tested for anti-*L. infantum* antibodies using an enzyme-linked immunosorbent assay (ELISA) [34] and for anti-*A. phagocytophilum* (MegaCor Diagnostik, Horbranz, Austria) and anti-*E. canis* (Biopronix Agrolabo, Scarmagno, Italy) antibodies by an indirect fluorescent antibody test (IFAT) (Fig. 1).

**Fig. 1:**
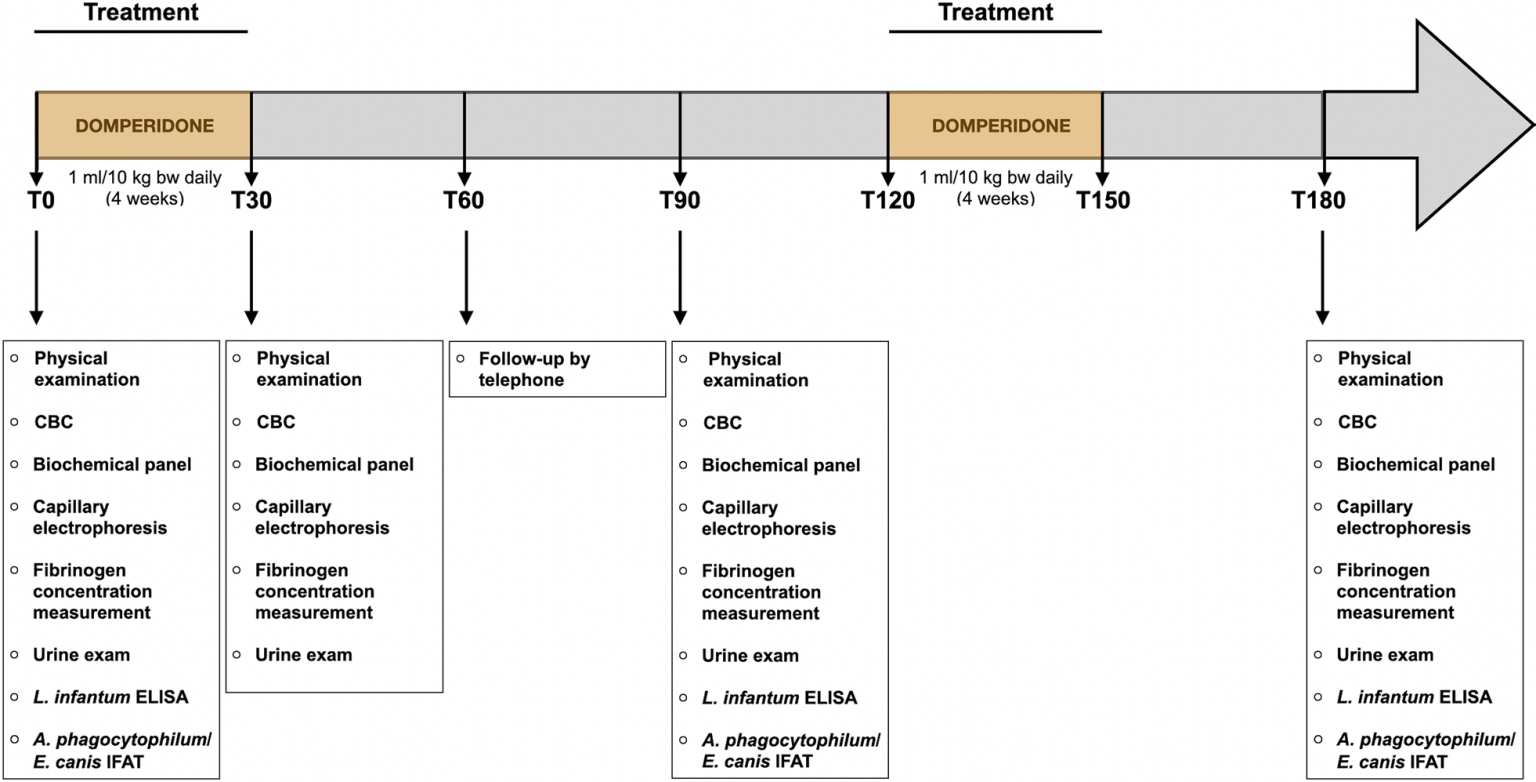
Schematic of the measurement time points (days), scheduled procedures and treatment protocol of the study. Abbreviations: CBC, Complete blood count; ELISA, enzyme-linked immunosorbent assay; IFAT, indirect fluorescent antibody test

Immediately after inclusion, dogs were treated with an oral suspension of domperidone (leisguard®) according to the following dosage and scheme: 1 ml/10 kg once daily for 4 weeks; 3 months without treatment; 1 ml/10 kg once daily for 4 weeks; finally, 1 month without treatment. The owners of the dogs checked product intake and the appearance of any side effects [35] throughout the administration period.

The enrolled dogs were followed up at 30 (T30), 60 (T60), 90 (T90) and 180 (T180) days after inclusion in the study, as shown in Fig. 1. At T30, T90 and T180, the dogs were given a complete physical examination, and onset or worsening of polyuria and polydipsia (PU/PD) (if present), onset or worsening of nausea or vomiting (if present), body condition score (BCS) (based on a scale of 1 to 5, with 3 being optimal) and body weight (kg) were recorded. At T60, the state of the dogs was followed-up by telephone, with the owner asked questions on the general clinical condition of the dog, onset or worsening of PU/PD (if present), onset or worsening of nausea or vomiting (if present), BCS evaluation and body weight (kg).

Statistical differences between SC, UPC, CRP, big Et-1, globulins, gamma globulins and anti-*L. infantum* antibody titers obtained at T0, T30, T90 and T180 were tested for significance by the Friedman test. A value of *P* < 0.05 was considered to be statistically significant. The statistical analyses were performed using Analyse-it (Analyse-it Software, Ltd., Leeds, UK).

Fourteen dogs were included in this study, as shown in Table 1. Five dogs were intact males and nine were females (six intact and three spayed), ranging from 27 to 118 months in age and weighing from 3 to 37 kg. Eight dogs were mixed breed, two were English cocker spaniel and one each was beagle, miniature pinscher, Labrador retriever and boxer. During the clinical trial, one dog (#10) was excluded after 27 days from T0 because of a surgical procedure due to gastric volvulus, while one dog (#5) was withdrawn and euthanized at T90 due to renal amyloidosis diagnosed by increased UPC and histologic findings from the post-mortem renal specimen biopsy (i.e. positive staining for Congo Red) (Table 1).

**Table 1:** Data on the 14 dogs enrolled in the study at T0

Dog ID no.	Inclusion (T0)						
	IRIS stage/NP, BP, P^b^	Creatinine (mg/dl) RI: 0.70–1.40	UPC^b^	*Leishmania infantum *ELISA (%)^c^	Globulins (g/dl) RI: 2.8–3.9	CRP (mg/dl) RI: 0.01–0.45	Big ET-1 (pg/ml)
1^a^	1/BP	1.07	0.21	Low (18.3**)**	4.1	0.16	2.4
2^a^	1/P	0.84	1.27	Medium (20.8)	3.6	0.05	2.1
3	1/P	0.74	4.5	High (33.9)	3.4	0.08	3.0
4	1/P	0.95	0.54	Low (14.7)	2.9	0.69	2.0
5^a^	1/P	0.55	1.69	Low (14.8)	3.1	0.03	NA
6	1/BP	1.12	0.23	High (32.0)	4.5	0.09	1.8
7	1/P	1.00	1.21	Medium (22.3)	3.8	0.29	1.6
8	2/P	2.37	1.74	High (30.5)	4.0	0.24	NA
9	1/BP	0.60	0.41	Medium (24.7)	4.0	0.41	1.8
10^a^	1/P	1.20	2.07	Low (17.5)	3.7	0.02	2.0
11	1/BP	1.12	0.21	Low (18.1)	4.3	0.19	1.9
12	1/BP	1.22	0.33	High (31.2)	4.5	0.12	1.8
13	1/P	1.10	0.60	Low (18.6)	4.1	0.32	NA
14^a^	1/BP	0.60	0.41	High (29.5)	4.0	0.41	2.1

All dogs were receiving treatment for chronic kidney disease for > 6 months

*Big ET-1* Big endothelin-1, *CRP* C-reactive protein, *ELISA* enzyme-linked immuosorbent assay,* NA* not applicable, *RI* reference interval, *T0* inclusion time point,* UPC* urinary protein/creatinine ratio

^a^Dogs excluded from the study at different time points

^b^IRIS stages: 1–4. NP, Non-proteinuric (UPC < 0.20); BP, borderline proteinuric (UPC = 0.21–0.50); P, proteinuric (UPC > 0.50) [31]

^c^Low 13–20%; medium 20.1–25%; high > 25%

Three dogs were excluded because of recurrence of active leishmaniosis (two dogs at T90 [#1, #14] and one dog [#2] at T180), diagnosed by clinical signs and/or pathological alterations, such as increased CRP and/or globulin values, hypalbuminemia and reduced albumin/globulin ratio, as well as direct identification of amastigotes in lymph node (*n* = 1) or bone marrow (*n* = 2) aspirates and increased anti-*L. infantum* antibody titer (Table 2).

**Table 2:** Comparison of creatinine, urinary protein/creatinine ratio, anti-*L. infantum* antibody titers, globulins, C-reactive protein and big endothelin-1 values of the three dogs which developed a recurrence of leishmaniasis during the trial course and were withdrawn at T90 (*n* = 2) and at T180 (*n* = 1)

Dog ID no.	T0						T90 (dog #1, #14)/T180 (dog #2)					
	Creatinine (mg/dl)	UPC	*L. infantum* ELISA (%)^a^	Globulins (g/dl)	CRP (mg/dl)	Big ET-1 (pg/ml)	Creatinine (mg/dl)	UPC	*L. infantum* ELISA (%)^a^	Globulins (g/dl)	CRP (mg/dl)	Big ET-1 (pg/ml)
1	1.07	0.21	Low (18.3**)**	4.1	0.16	2.4	0.93	2.10	High (30.7)	8.6	7.04	2.8
2	0.84	1.27	Medium (20.8)	3.6	0.05	2.4	0.69	2.16	High (34.3)	5.4	0.15	2.4
14	0.60	0.41	High (29.5)	4.0	0.41	2.1	0.60	1.61	Medium (23.8)	5.1	1.16	3.6

T90, T180 90 and 189 days after inclusion day/start of initial treatment

^a^Low 13–20%; medium 20.1–25%; high > 25%

All nine dogs that completed the entire pilot study showed a statistically significant reduction in SC (*χ*^2^ = 9.1, *df* = 3, *P* = 0.028) (Fig. 2), but not in UPC (*χ*^*2*^ = 6.43, *df* = 3, *P* = 0.092). Furthermore, even the three dogs that developed a recurrence of leishmaniosis during the trial course and were withdrawn at T90 (*n* = 2) and at T180 (*n* = 1) maintained a stable serum creatinine level (Table 2).

**Fig. 2:**
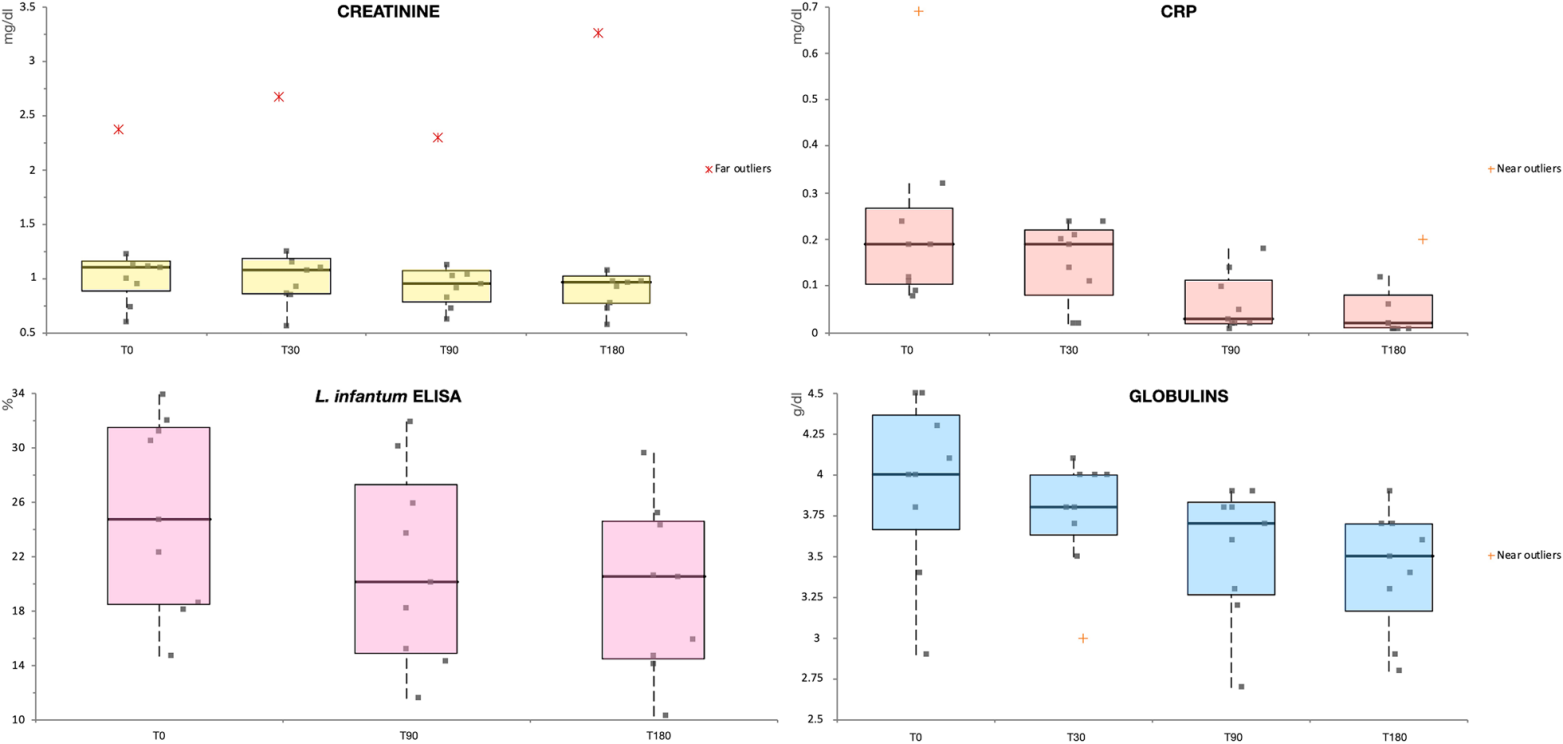
Box-plot graph of creatinine, C-reactive protein (CRP), globulins and anti-*L. infantum* antibody titers obtained at T0, T30, T90 and T180. Values were tested for significance by the Friedman test

During the study period, there was a significant reduction in all dogs of antibody titers for *L. infantum* (*χ*^2^ = 9.56, *df* = 2, *P* = 0.008), globulins (*χ*^2^ = 11.08, *df* = 3, *P* = 0.011) and gamma globulins (*χ*^2^ = 12.38, *df* = 3, *P* = 0.006) (Fig. 2). The Friedman test showed a statistically significant reduction in CRP during the study period (*χ*^2^ = 16.7, *df* = 3, *P* = 0.001), but not in big ET-1 (*χ*^2^ = 2.04, *df* = 3, *P* = 0.563), when values at T0, T30, T90 and T180 were compared (Fig. 2). No further alterations in the parameters included in the biochemical panel were recorded (e.g. electrolytes) during the study period. In addition, all enrolled dogs had an inactive urinary sediment and urine specific gravity (USG) within normal limits (1015–1045).

All nine dogs that completed the study tested negative to anti-*A. phagocytophilum* and anti-*E. canis* antibodies by IFAT at T0, T90 and T180. No side effects related to domperidone administration were reported [34].

This pilot study provides preliminary data on the efficacy of domperidone (leisguard®) in improving renal function (SC) in dogs with leishmaniosis affected by CKD. The data from the present study also confirm previous published results on the ability of this drug to significantly reduce anti-*L. infantum* antibody titers [19] while showing for the first time a potential effect in improving clinical and laboratory values for globulins, gamma globulins or other inflammatory markers, such as CRP.

High levels of antibodies are associated with a high parasitic burden [36]. Consequently, the reduction of antibody titers is related to the ability of domperidone to indirectly cause a regression of antigenic stimulation by *L. infantum*. Indeed, domperidone activates dogs’ cell-mediated immune response by increasing the levels of prolactin in blood, with prolactin considered to be a proinflammatory lymphocyte-derived cytokine [19, 37, 38]. A prolactin-mediated stimulation of the Th1 immune response causes release of interleukin (IL)-2, IL-12, interferon (INF)-γ and tumor necrosis factor (TNF)-α, which leads to activation of natural killer cells and macrophages followed by a decrease in CD4 + Th2 subsets and TNF-β [39–41]. According to this scenario, domperidone could promote *L. infantum* load control and prevent progression of the disease. Interestingly, the reduction in antibody titer in the enrolled dogs shown by the ELISA occurred not only in the presence of a initial antibody titer (high, medium or low) but also throughout the sand fly season in the study area (i.e. from May to November; [42]) during which time antibody levels generally tend to rise [43]. It is noteworthy that the degree of reduction in humoral response could be affected by the response of the individual dog [44].

The study population showed a statistically significant reduction in SC (*χ*^2^ = 9.1, *df* = 3, *P* = 0.028). The renal effects of domperidone in leishmaniotic dogs with CKD can be considered to be a result of either a “direct” or “indirect” intrarenal action. According to [28], the “direct” action can be associated to the ability of specific DA_2_ receptor antagonists being able to increase GFR, RPF and the filtration fraction, thereby potentially promoting renal filtration and the maintenance of adequate SC levels. An “indirect” action of domperidone on the kidney due to the improvement of CanL-related parameters, such as a significant reduction in antibody titers for *L. infantum* (*χ*^2^ = 9.56, *df* = 2, *P* = 0.008), globulins (*χ*^2^ = 11.08, *df* = 3, *P* = 0.011) and gamma globulins (*χ*^2^ = 12.38, *df* = 3, *P* = 0.006), could not be excluded. Indeed, the major cause of glomerular pathology in CanL is represented by the deposition of preformed CIC at different levels of the glomerular unit, triggering localized inflammation [12]. Domperidone might reduce the formation of CIC due to the decrease of antigenic stimulation, obtained by activating the phagocytic polymorphonuclear cells involved in the innate immune response and the reduction of antibody production [19, 20]. Therefore, SC reduction might be associated to a reduction of CIC, as previously described, where SC level was significantly correlated to CIC levels during CanL [9, 45].

Although outside the main aims of the study, attention should be paid to the three dogs that developed a recurrence of leishmaniosis during the trial course, presenting an increase in anti-*L. infantum* antibody titers, globulins, CRP and UPC (Table 2). All three dogs maintained a stable SC level despite clear evidence of a relapse of leishmaniosis and in contrast with previously published data that demonstrated a strict relationship between CIC and SC level [9, 45], thus making also a direct intrarenal effect of domperidone more likely.

On the other hand, although it is possible to detect a free light-chain proteinuria in absence of structural or functional renal derangement in dogs with leishmaniosis [46], values of proteinuria usually mirror the extent of the glomerular kidney damage due to the deposition of CIC [11, 12]. Therefore, a treatment which would be able to improve renal perfusion, such as domperidone treatment, would be unlikely to have a direct impact on proteinuria. Indeed, none of the enrolled dogs showed a statistically significant reduction in UPC. Also to be considered is the possibility that domperidone, by ameliorating RPF, might determine intraglomerular hypertension, thus conditioning the grade of proteinuria [47].

In this study globulins, gamma globulins and CRP showed a significant decrease over time. Although pre-treatment CRP values were within normal limits in all enrolled dogs (Table 1), the significant reduction in this APP suggests that some activation of inflammatory/immune responses occurred secondary to exposure to the parasite. At the same time, the CRP values at T0 support the hypothesis that the enrolled dogs did not have an active infection and that spreading of the parasite typically occurs only in sick dogs [2, 44], as occurred in the three dogs withdrawn from the study (Table 2). This result regarding CRP substantially confirms findings from previous studies on the short-term effect on domperidone on CRP concentration: after a transient increase in the first days of treatment consistent with a domperidone-induced increased metabolism of phagocytes, there was a trend to a decreased CRP concentration towards the end of the first month of treatment [19, 44].

The lack of a significant decrease of Big-ET1 during the study period likely depends on the very low level of this analyte recorded at T0, which hampered the possibility to observe further decreases during the follow-up. Big-ET1 is known to increase in response to hypertension associated with advanced stages of CKD [48]. Therefore, the very low level of this APP can be related to the fact that enrolled dogs, despite the possible activation of the inflammatory or immune responses mentioned above, were not in an advanced kidney disease stage.

The main limitations of the present study are the absence of a CanL-positive control group not treated with domperidone and the small sample size due to the application of strict inclusion criteria. The pilot nature of the study and its realistic constraints (such as budget limits) also affected the number of dogs that could be enrolled. Moreover, dogs enrolled in the study did not undergo renal biopsy; thus, there was no complete characterization of the renal damage or a final histopathological classification according to current guidelines [2, 31]. However, the characteristics of the enrolled animals, such as clinical stability for CKD, made a renal biopsy unnecessary according to current indications [49, 50]. Finally, despite these limitations, the strict inclusion criteria, the 180-day-long follow-up of the included cases and the statistically significant results of the present study may provide interesting information on the clinical application of domperidone (leisguard®) for the management of CanL.

In conclusion, this pilot study provides preliminary results on the ability of domperidone to improve kidney function by reducing anti-*L. infantum* antibody titers, globulins, gamma globulins and CRP in dogs with leishmaniosis affected by CKD. However, further studies are necessary to test the efficacy of domperidone in improving the kidney function of dogs with leishmaniosis in advanced stages of CKD and to elucidate the effect of this drug in *L. infantum*-negative dogs affected by renal insufficiency.

## Data Availability

The datasets generated and/or analyzed during the current study are available from the corresponding author on reasonable request.

## Abbreviations

APPs: Acute-phase proteins
BCS: Body condition score
big ET-1: Big endothelin-1
CanL: Canine leishmaniosis
CBC: Complete blood count
CIC: Circulating immune complexes
CKD: Chronic kidney disease
CRP: C-reactive protein
DA_2_: Dopamine 2
GFR: Glomerular filtration rate
IFN: Interferon
IL: Interleukin
PU/PD: Polyuria and polydipsia
RPF: Renal plasma flow
SC: Serum creatinine
TFN: Tumor necrosis factor
UPC: Urinary protein/creatinine ratio
USG: Urine specific gravity
